# Bivalve Omics: State of the Art and Potential Applications for the Biomonitoring of Harmful Marine Compounds

**DOI:** 10.3390/md11114370

**Published:** 2013-11-01

**Authors:** Victoria Suárez-Ulloa, Juan Fernández-Tajes, Chiara Manfrin, Marco Gerdol, Paola Venier, José M. Eirín-López

**Affiliations:** 1 Chromatin Structure and Evolution (CHROMEVOL) Group, Department of Biological Sciences, Florida International University, North Miami, FL 33181, USA; E-Mail: msuarezu@fiu.edu; 2 Wellcome Trust Center for Human Genetics, University of Oxford, Oxford OX3 7BN, UK; E-Mail: jfertaj@well.ox.ac.uk; 3 Department of Life Sciences, University of Trieste, Trieste 34127, Italy; E-Mails: cmanfrin@units.it (C.M.); mgerdol@units.it (M.G.); 4 Department of Biology, University of Padova, Padova 35121, Italy; E-Mail: paola.venier@unipd.it

**Keywords:** marine invertebrates, omics, bioinformatics, pollution, biomonitoring, biotoxins, heavy metals, PAHs

## Abstract

The extraordinary progress experienced by sequencing technologies and bioinformatics has made the development of omic studies virtually ubiquitous in all fields of life sciences nowadays. However, scientific attention has been quite unevenly distributed throughout the different branches of the tree of life, leaving molluscs, one of the most diverse animal groups, relatively unexplored and without representation within the narrow collection of well established model organisms. Within this Phylum, bivalve molluscs play a fundamental role in the functioning of the marine ecosystem, constitute very valuable commercial resources in aquaculture, and have been widely used as sentinel organisms in the biomonitoring of marine pollution. Yet, it has only been very recently that this complex group of organisms became a preferential subject for omic studies, posing new challenges for their integrative characterization. The present contribution aims to give a detailed insight into the state of the art of the omic studies and functional information analysis of bivalve molluscs, providing a timely perspective on the available data resources and on the current and prospective applications for the biomonitoring of harmful marine compounds.

## 1. Introduction

Marine invertebrates constitute the largest group of macroscopic species in the sea [1]. Among them, bivalve molluscs stand out not only for their fundamental role in the marine ecosystem, but also for their commercial value in aquaculture industry [2,3]. Additionally, this group of organisms displays key features legitimizing their application as sentinel organisms for the biomonitoring of harmful compounds, particularly in coastal and estuarine areas, including: ubiquitous distribution, easy accessibility, filtering lifestyle, as well as strong resistance to a wide range of pollutants [4,5,6,7,8,9,10]. Bivalves have been traditionally tested for biomonitoring purposes [11,12,13], often following physiological or biochemical approaches. Yet, it was not until very recently that integrative omic approaches have been implemented in the study of marine bivalves, primarily due to the recent advances in sequencing technologies and the substantial reduction in the associated costs. Nonetheless, the characterization of bivalve genomes is still challenging given the lack of reference assemblies as well as the presence of specific sequence features such as high density of repetitive regions and increased levels of polymorphism [7,14]. Consequently, alternative approaches tackling the study of specific genome regions using Next Generation Sequencing (NGS) platforms have been implemented, most notably *de novo* 454-pyrosequencing of transcriptomes [5,15,16,17].

**Figure 1:**
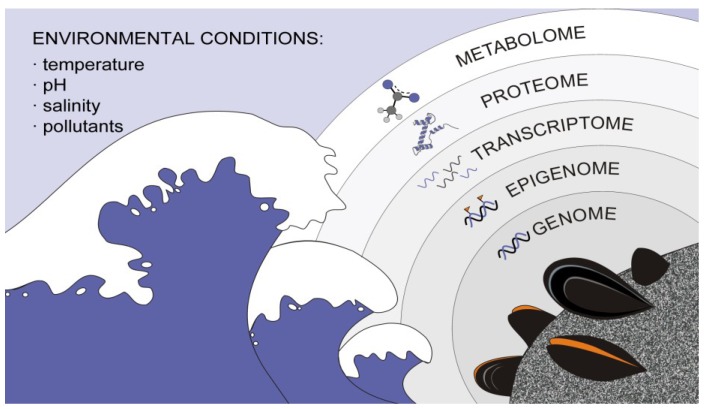
Integrative omic studies constitute a powerful tool in addressing the links between environmental conditions, harmful effects and associated responses in marine bivalves. Environmental conditions affect different levels, starting from the genome and the state of the chromatin (epigenome). Changes on these levels influence gene expression and the pool of expressed mRNAs (transcriptome), which in turn has an obvious effect on protein synthesis (proteomics). The regulation of all these systems also produces modifications in the set of small metabolites produced by an organism (metabolome). Overall, the intricate interconnection among the different omes requires a holistic integrative approach in order to understand how organisms respond to changes in the surrounding environment.

So far, the omic study of bivalves has been mainly focused towards the characterization of genomes, transcriptomes and proteomes, although not necessarily in this specific order. In fact, pioneer omic studies in bivalves were eminently based on transcriptomes, helping to set up the foundations for subsequent proteomic and genomic studies [18,19,20,21,22]. Metabolomic and epigenomic characterization of bivalves, on the other hand, still constitute emerging disciplines that would complete the necessary framework for integrative approaches (Figure 1). Ongoing interest in omic studies of bivalves is mirrored by recent publications of draft and complete genome sequences for two oyster species, the pearl oyster *Pinctada fucata* [14] and the Pacific oyster *Crassostrea gigas* [7]. In addition, major efforts are being carried out in the midst of the current “omics rush” to push bivalve omics forward, as illustrated by the characterization of diverse transcriptomes and proteomes in other bivalves [16,23,24,25,26,27,28,29]. Within this scenario, the present work aims to put together a timely and comprehensive review of the state of the art in the omic analysis of bivalve molluscs, with emphasis on currently available web-accessible molecular data resources and their potential applications for the biomonitoring of harmful marine compounds.

## 2. Leading Edge on Bivalve High-Throughput and NGS Data Analysis

### 2.1. Bivalve Genomes

As mentioned earlier, although closely followed by the still gapped genome assembly of *P*. *fucata* [14], the only bivalve genome currently considered as complete belongs to the Pacific oyster *C*. *gigas* [7]. Yet, why is there only one single bivalve genome completely sequenced, despite their commercial and biologic importance in the “omics” era? It seems that the repetitive organization of the non-coding fraction in bivalve genomes, as well as their size, truly represent a challenge for their *de novo* sequencing and for the efficient assembly of these repeated pieces of information. On one hand, the estimated C-value of most bivalve genomes ranges between 0.5 and 2.0 pg (*C*. *gigas* genome is approximately 558 Mb in size) [30], over 10 times the size of the most well studied invertebrate model organisms (e.g., *Drosophila melanogaster*, *Caenorhabditis elegans*, *etc*.). On the other hand, it has been reported that one single satellite repetitive DNA sequence might comprise 0.63% of the genome of the blue mussel *Mytilus edulis* [31] and that 30% of the genome of the *C*. *gigas* is repetitive DNA [7]. The complexity of bivalve genomes is mirrored by the efforts carried out in the pearl oyster *P*. *fucata* genome sequencing project, where a considerable sequencing coverage (~40 fold) was necessary in order to produce a draft genome [14]. The development of specific sequencing and assembly methodologies, such as those developed during the sequencing of the *C*. *gigas* genome on which fosmid pooling and hierarchical assembly were used [7], are expected to improve the development of new bivalve genome projects.

### 2.2. Bivalve Transcriptomes

Initial transcriptomic studies based on homology cloning in bivalves were progressively complemented with the analysis of genes differentially expressed in response to different pollutants and pathogens, based on different technologies such as cDNA libraries, Suppression Subtractive Hybridization libraries (SSH) and microarrays [5,6,8,9,16,19,20,32,33,34,35,36,37,38] (see Section 4 for details). The development of cDNA and SSH libraries has led to a significant increase in the number of Expressed Sequence Tags (ESTs) in databases, constituting the basis for DNA microarray technology. Microarrays have been primarily used in mussels to study the large-scale transcriptional response to different environmental stress factors [13,18,19,34,39,40]. Nowadays, the combination of microarray and NGS technologies is significantly speeding up *de novo* gene discovery and microarray design [41], allowing transcriptomic analyses of non-model organisms [42] including bivalves [16,25,29,43,44]. Additionally, the RNA-seq approach to transcriptome profiling is becoming an appealing alternative to the DNA microarray analysis also in bivalves [45]. RNA-seq provides a far more precise measurement of transcript levels than other methods, delivering unbiased and unparalleled gene expression information. Transcriptome assembly poses specific challenges of its own given that, unlike genomes, the number of sequenced reads pertaining to different transcripts can vary over several orders of magnitude due to differences in expression levels [42]. Consequently, sequencing coverage is susceptible to be heterogeneous throughout the whole transcriptome (*i*.*e*., higher coverage levels of highly expressed transcripts) requiring transcript normalization before data analysis.

### 2.3. Bivalve Proteomes

Changes in cell phenotype can be fully appreciated only when transcripts are translated into proteins [46]. The field of proteomics has flourished hand in hand with the advancement in techniques of protein separation and identification, mainly two-dimensional gel electrophoresis and multidimensional Liquid Chromatography combined with Mass Spectrometry (LC-MS) technologies [47]. While gel-based techniques display serious limitations referred to proteome coverage, gel-free techniques as LC-MS are considered fast and low-cost high-throughput approaches [48]. However, the large-scale application of shotgun gel-free proteomic methods in bivalves remains hampered by the relative scarcity of genomic data in public repositories, necessary for automated protein identification [4]. So far, the proteomic study of bivalves pointed to the identification of biomarkers of aquatic pollution [49,50,51] and general gene/protein expression profile studies [52,53,54]. In addition, proteome investigation is currently being used to study the bivalve response to different sources of environmental stress such as the study of proteomic changes in response to ocean acidification [55,56].

### 2.4. Bivalve Metabolomics

Metabolomics is a recently developed omic field focused on the integral study of the metabolic profile of a cell or system, especially low molecular-weight metabolites (<1000 Da) regarded as fingerprints of specific biological processes. A major advantage of this approach is that it does not make any assumptions about the relevance of the different metabolites and does not require previous knowledge on the genome of the organisms studied [4]. On the other hand, a major criticism to the application of metabolomics in the biomonitoring of toxicity is the difficulty in correlating different sources of toxicity with changes in specific metabolites [57]. Among bivalves, the study of the mussel metabolome has been used to assess the effect of heavy metal contamination [58,59], to discriminate sex specific metabolites and to understand the mode of action of pesticides like atrazine and lindane [60]. Additionally, the metabolome of the manila clam *R*. *phillipinarum* was studied to evaluate exposure to heavy metals [61,62,63] and benzo(a)pyrene [64].

### 2.5. Bivalve Epigenomics

The epigenomic analysis of bivalve genomes seeks a global profiling of epigenetic marks and chromatin structure using high-throughput methods [65], constituting a prospective field of great interest both in terms of basic and applied research. Besides helping ascertain the evolution of the several layers of complexity regulating gene expression during development [66], the characterization of genome-wide patterns of chromatin reorganization in response to environmental stress will provide researchers with a promising approach to detect and quantify levels of different marine compounds by using bivalves as sentinel organisms. Even though these objectives are ambitious, the epigenetic relevance of DNA methylation in oysters [67], the characterization of histones and histone variants in several bivalve molluscs [68,69,70,71] as well as the characterization of their expression profiles and posttranslational modifications in response to the marine biotoxin Okadaic Acid (OA) have been reported [5,72].

## 3. Resources for the Study of Bivalve Omic Data

### 3.1. General Resources

**Figure 2:**
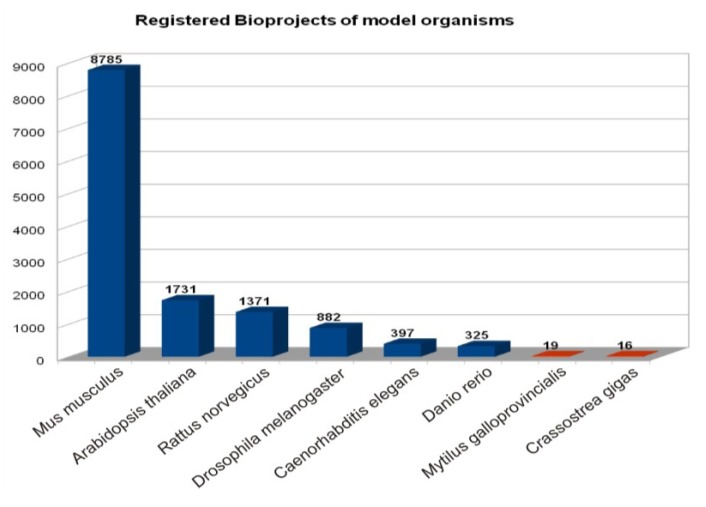
Number of bioprojects registered at the NCBI database comparing traditional model organisms with two upcoming bivalve model organisms (the mussel *M*. *galloprovincialis* and the oyster *C*. *gigas*).

During the genomic and post-genomic era, the scientific community has witnessed the swiftest and largest expansion of public repositories of molecular data, especially regarding humans and traditional model organisms. However, only a small number of genome projects focused on bivalves are currently registered at the National Center for Biotechnology Information (NCBI) database (Figure 2), including 2 freshwater species and 17 marine species [73]. Indeed, all the entries belonging to bivalves registered in the NCBI Reference Sequence (RefSeq) Database remain limited to mitochondrial genomes [74]. On the contrary, a rich variety of transcriptomic datasets are currently being produced and submitted to public repositories (Gene Expression Omnibus, GEO [75]), providing valuable knowledge encompassing straightforward environmental applications.

Nowadays, the molecular databases supported by NCBI play a leading role given their standardization and durability. In addition to the well known Genbank [76] and GEO databases [77,78], the blossoming of high-throughput technologies has unfolded new specialized subdivisions such as the NCBI Sequence Read Archive (SRA [79]) and the Transcriptome Shotgun Assembly database (TSA [80]). In order to submit data to SRA or TSA, a Bioproject must be previously registered along with a description of the basic features and global research aim of the datasets, thus facilitating cross referencing (Figure 2). A summary of Bioprojects, registered Genome projects and SRA datasets currently available on marine bivalves is shown in Table 1.

**Table 1:** High-throughput data registered in public repositories of the NCBI.

Species	Bioproject		Genome	SRA Datasets			
	Number	Type		Total	454	Illumina	AB SOLiD
*Alasmidonta varicosa*	1	transcriptome/gene expression	YES (no data)	1	1	-	-
*Arctica islandica*	1	genome	YES (no data)	12	12	-	-
*Argopecten irradians*	3	transcriptome/gene expression	-	1	1	-	-
*Bankia setacea*	1	genome	YES (no data)	1	1	-	-
*Bathymodiolus azoricus*	1	transcriptome/gene expression	YES (no data)	1	1	-	-
*Chlamys farreri*	1	transcriptome/gene expression	-	-	-	-	-
*Chamelea gallina*	-	-	-	1	1	-	-
*Crassostrea angulata*	1	transcriptome/gene expression	-	-	-	-	-
*Crassostrea gigas*	16	1 genome, 15 transcriptome/gene expression	YES (scaffold or contigs status)	159	2	155	2
*Crassostrea hongkongensis*	1	proteome	-	-	-	-	-
*Crassostrea virginica*	3	transcriptome/gene expression	-	-	-	-	-
*Ennucula tenuis*	-	-	-	1	1	-	-
*Glossus humanus*	1	exome	YES (no data)	-	-	-	-
*Hyriopsis cumingii*	1	transcriptome/gene expression	-	1	1	-	-
*Laternula elliptica*	1	transcriptome/gene expression	-	3	3	-	-
*Macoma balthica*	-	-	-	3	3	-	-
*Mercenaria mercenaria*	1	transcriptome/gene expression	YES (no data)	2	-	-	2
*Meretrix meretrix*	1	transcriptome/gene expression	-	1	1	-	-
*Mizuhopecten (Patinopecten) yessoensis*	1	transcriptome/gene expression	-	2	2	-	-
*Mya arenaria*	2	transcriptome/gene expression	YES (no data)	-	-	-	-
*Mytilus californianus*	4	transcriptome/gene expression	-	-	-	-	-
*Mytilus edulis*	-	-	-	44	44	-	-
*Mytilus galloprovincialis*	19	transcriptome/gene expression	-	12	6	6	-
*Nodipecten subnodosus*	-	-	-	2	2	-	-
*Nucula nitidosa*	-	-	-	1	1	-	-
*Ostrea lurida*	2	transcriptome/gene expression	YES (no data)	1	-	1	-
*Pinctada fucata*	1	transcriptome/gene expression	YES (no data)	10	7	3	-
*Pinctada margaritifera*	2	transcriptome/gene expression	YES (no data)	1	1	-	-
*Pinctada maxima*	7	transcriptome/gene expression	-	1	1	-	-
*Pteria penguin*	1	transcriptome/gene expression	YES (no data)	-	-	-	-
*Ruditapes decussatus*	3	transcriptome/gene expression	-	-	-	-	-
*Ruditapes philippinarum*	6	transcriptome/gene expression	-	28	2	26	-
*Solemya velum*	-	-	-	2	1	1	-
*Spisula solidissima*	1	genome	YES (no data)	-	-	-	-
*Tegillarca granosa*	1	transcriptome/gene expression	-	1	1	-	-
*Yoldia limatula*	-	-	-	1	1	-	-

Projects are registered in the Genome specific database and become graded by curators with status symbols detailing the type and amount of data provided. So far, only *C*. *gigas* has acquired the status of scaffold while the others are only registered with no data yet submitted (see NCBI Genome [73]). Along with genomic data, the *C*. *gigas* sequencing project has provided a large number of SRA submissions, including RNA-seq data from gene expression experiments under environmental stress [7]. Although no genome sequencing data is yet available in mussels, species from the genus *Mytilus* follow in numbers of registered projects and submitted datasets, especially in the case of *M*. *galloprovincialis* [81]. Gene expression profiling using microarray technologies is often carried out in these organisms by means of tailor-made microarray platforms [6,13,18,19,21,23,34,35,39,40,82,83,84,85,86,87,88,89]. RNA-Seq expression studies, on the other hand, are still hampered by the lack of reference genomes. Overall, oysters, mussels and clams attract most of the scientific attention given their high commercial value and their potential applications as sentinel organisms in marine pollution biomonitoring [90].

### 3.2. Specialized Resources

#### 3.2.1. Databases and Knowledge Repositories

Although most molecular information related to bivalve molluscs is stored in the repositories detailed in the previous section, a number of specialized databases have become publicly available during the last years (see Table 2). For instance, the Marine Genomics Project [91] comprises ESTs and microarray data from marine organisms in a broad sense, although most recent databases aim to extend the molecular knowledge to specific group of species. Within this context, the genome draft of the pearl oyster *Pinctada fucata* (version 1.0) has been made publicly available through a specific genome browser [14,92]. Similarly, repositories such as the Mytibase [93] represent useful resources for the transcriptomic study of the mussel *Mytilus*, providing large-scale ESTs with critical relevance for developing microarray platforms aimed to the biomonitoring of marine pollution. ESTs have been also put together for other bivalve species such as the clams *Ruditapes philippinarum* in the RuphiBase [35] and *Chamelea gallina* in the ChamaleaBase [27], the mussel *Bathymodiolus azoricus* in the DeepSeaVent database [33], as well as the Pacific oyster *C*. *gigas* in the GigasDatabase [94]. From a functional perspective, the CHROMEVALOA database [5] constitutes a resource aimed to provide a specific platform for the evaluation of OA contamination in the marine environment based on the chromatin-associated transcriptome of the mussel *Mytilus galloprovincialis* (transcripts involved in chromatin structure and metabolism, differentially expressed in response to OA). The future coordination of the aforementioned resources could constitute a cross-referenced network providing all necessary information about the transcriptional features of the environmentally relevant class of Bivalvia.

**Table 2:** Summary of the transcriptomic databases specialized in bivalves.

Database		*Organism*/# Sequences	*Tissues*/URL
Species-centered			
	Mytibase	*M*. *galloprovincialis* 7112	*Digestive gland*, *gills*, *hemocytes* [95]
	GigasDatabase	*C*. *gigas* 29745	*Digestive gland*, *gills*, *gonad*, *hemocytes*, *mantle-edge*, *muscle* [96]
	RuphiBase	*R*. *philippinarum* 32606	*Mixed tissues* [97]
	ChameleaBase	*C*. *gallina* 36422	*Muscle* [98]
	DeepSeaVent	*B*. *azoricus* 35903	*Gills* [99]
Functionally-centered			
	Chromevaloa	*M*.*galloprovincialis* 14408	*Digestive gland* [100]

#### 3.2.2. Array Technology

So far, most gene expression studies carried out on bivalves have relied on microarray technologies so far, with RNA-seq projects still scarcely represented. Platforms are usually designed and built *ad hoc* with specific oligo probes of one or more organisms of interest. The mussel *M*. *galloprovincialis* is the target organism in 5 specific platforms registered in GEO, focused on the transcriptional response of mussels exposed to a number of seawater pollutants [19,89] as well as to the biotoxin OA [13] using Mytarray 1.0 (accession GPL1799). The upgraded Mytarray 1.1 (accession GPL10269), allows gene expression studies during annual cycles and discriminates between sexes [82]. Additionally, the Mussel Immunochip (accession GPL10758) and HMS/SomeroLab-Mytilus-105k (accessions GPL9676 and GPL11156) have also been used to assess the effects of different environmental conditions such as infectious processes [34] or physico-chemical stress [39]. Furthermore, a robust *M*. *galloprovincialis* microarray is currently being developed to study environmentally relevant biotoxins [101]. Similarly, *C*. *gigas* has been also the subject of up to 6 specific microarray platforms registered in GEO, including 1 with mixed probes from *Crassostrea virginica*, aimed to assess coastal pollution levels [37]. Although the development of specific microarrays is very common now in oysters and clams [35,87], the RNA-seq approach is progressively replacing DNA array analysis in gene expression studies, as illustrated by the cases of *C*. *gigas* [7] and *M*. *galloprovincialis* [45].

## 4. Bivalve Omic Approaches for the Biomonitoring of Marine Compounds

**Figure 3:**
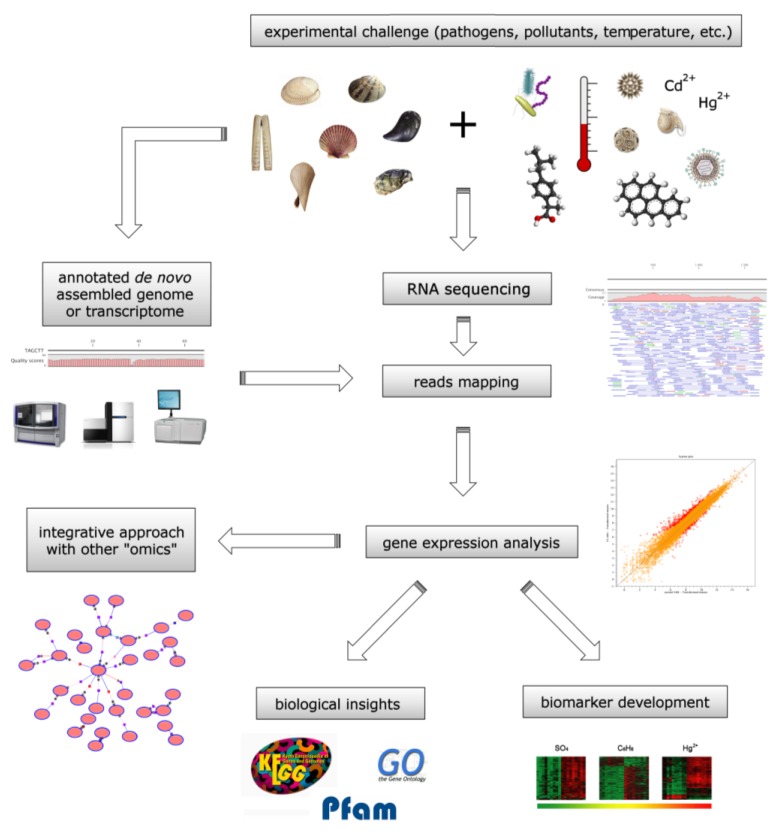
Process flow diagram of a NGS-based transcriptome analysis and potential applications for environmental biomonitoring. Specimens are subject to the desired experimental challenge and, upon collection, total RNA is extracted and sequenced using the technology of choice. Depending on the pre-existing genomic resources available for the organism of interest, sequencing reads are then mapped to an annotated reference genome or transcriptome to obtain read counts for each gene, and finally converted into digital gene expression data. The comparison of the gene expression profiles obtained from treated and control samples can lead to biological insights on the transcriptional response to the experimental stimulus, to the identification of potential gene expression biomarkers and also allows integrative analyses with other omic approaches (*i*.*e*., proteomics, metabolomics, *etc*.).

The application of omic approaches based on high-throughput and NGS molecular data constitutes a very powerful tool in deciphering the molecular mechanisms underlying the response and adaptation of bivalves to environmental changes (Figure 3). Nowadays, the analysis of gene expression profiles is helping define new metrics complementing traditional chemical and biomarker measures, redefining the surveillance of coastal water pollution. On one hand, SSH and microarray techniques continue to provide important information concerning differential expression of specific genes in response to different sources of stress in the surrounding environment. On the other hand, high-throughput sequencing of transcriptomes (RNA-seq) is progressively adding further depth and new details on bivalve biology.

### 4.1. Bivalve Transcriptomes as Biomonitoring Tools

The dynamic transcriptional response to fluctuations in environmental factors has the potential to reveal transitory adjustments, irreversible functional deficits and taxon-specific adaptive features of the organisms. For instance, the study of transcript signatures has revealed subtle species-specific transcriptomic differences between the mussels *Mytilus galloprovincialis* and *M*. *trossulus* [40]. Additionally, transcriptomic profiles have also been addressed in the oyster *Crassostrea virginica* using chemical measures and DNA microarray analyses both on gill and digestive gland samples. In this case, progressive computational data testing confirmed the reliability of the DNA microarray metrics and provided insights on the mechanisms of responses to temperature and pH in oysters [37]. Concurrently, the construction of SSH libraries led to the identification of putative biomarkers involved in the response to several stress factors relevant for aquaculture, including high temperature [39,102], hypoxia [103] and pathogen infection [104,105].

Transcriptomic studies have helped identify specific groups of genes involved in the response and adaptation of bivalves to external conditions. This is the case of studies suggesting that genes involved in defense and the innate immune response play a pivotal role as determinants of the resistance to summer mortality in *C*. *gigas* [83,85]. Indeed, sequencing and data mining of ESTs are essential steps for the comparative identification of molecules and related pathways of response to specific stimuli [32,34,36,86,94] and this task is greatly facilitated by the availability of high throughput sequencing technologies yielding unprecedented amounts of sequence data. RNA-seq has also been used to identify genes involved in the development of *Crassostrea angulata* and *Meretrix meretrix* [26,28]. As illustrated, the combination of molecular data with traditional physiological and population studies provides a new framework for the management of livestock under naturally- or anthropically-driven stress conditions, improving both open water and hatchery aquaculture systems [25,27].

### 4.2. Toxin Biomonitoring during Harmful Algae Blooms

Among marine compounds, Harmful Algae Blooms (HABs) cause deleterious effects not only in natural populations of bivalves (and other organisms), but also on human health and economy. So far, three different studies using the Mediterranean mussel *M*. *galloprovincialis* as model organism have tried to tackle the effects of marine biotoxins on bivalves from an omic perspective. In a first study, the effects of the accumulation of the biotoxin OA in mussels over a 35 day exposure period were studied by using a cDNA microarray, resulting in the identification of several transcripts as candidates of OA-stress markers [13]. Although most identified sequences could not be linked to known metabolic pathways correlated to OA biotransformation, the up-regulation of several stress-related proteins involved in apoptosis, proteolysis and cytoskeleton destabilization, suggested a harmful effect of OA in mussels. In a second study, the characterization of the chromatin-associated transcriptome of mussels exposed to OA was carried out, now available in the CHROMEVALOA database [5]. This work lays the foundations for the study of chromatin-related transcriptome changes potentially involved in the response to OA. Indeed, this approach permitted the identification of a number of genes whose expression was significantly influenced by OA, revealing potential sensitive biomarkers for OA genotoxicity tests. A third study, currently in progress, investigates the molecular mechanisms underlying the response to the accumulation of paralytic shellfish toxins produced by the dinoflagellate *Alexandrium minutum* in the digestive gland of mussels over a period of 5 days. Preliminary results suggest that even though negligible effects on gene expression seem to be produced by biotoxin contamination, a few potential biomarkers of contamination were identified [106].

### 4.3. Evaluation of the Harmful Effects of Anthropic Pollutants

Compounds present in the sea as a direct consequence of human activities still represent an issue of great concern, especially in heavily anthropized coastal regions. These compounds include pesticides and drugs, polycyclic aromatic compounds, heavy metals and many other chemicals resulting from industrial and urban settlements, conveyed in wastewaters and rivers and finally entering the oceans. Since the molecular effects of most chemical compounds on marine organisms and communities are still poorly understood, the development of studies based on omic approaches could improve the evaluation of their impact and provide a more robust basis for biomonitoring programs. With this in mind, recent studies using SSH libraries have identified potential biomarkers of exposure to emerging PAHs in the digestive gland of the mussel *M*. *edulis*, namely the carcinogenic compounds styrene [107] and benzo[*a*]pyrene [21]. Additionally, the still poorly understood adverse effects of diesel fuel have been addressed in *Crassostrea brasiliana* [9] whereas the generation of SSH libraries in bivalves has clarified the molecular effects of other anthropic pollutants such as agricultural pesticides [8,22] or cadmium from industrial or urban settlements wastewaters [20]. Similarly, microarray technologies have been used to investigate the effects of exposure to copper and organophosphate pesticides in *M*. *galloprovincialis* (BioProject PRJNA178507), revealing a interference of the pesticide Chloropyrifos with natural estrogens such as 17β-estradiol [20]. Interestingly, the same microarray platform used in studies of OA exposure [13] has also been applied to evaluate the synergistic effects of pesticide and heavy metal exposure in *M*. *galloprovincialis* [108].

Other examples of transcriptomic approaches to the study of anthropic compounds include microarray-based studies of Tributyltin, a chemical biocide used in marine antifouling paints that, in addition to causing shell abnormalities, also affects biomineralization pathways in the oyster *Pinctada maxima* (BioProject PRJNA114601). Similarly, the expression profile of the digestive gland of the manila clam *Ruditapes philippinarum* was studied by using an Agilent Oligo Microarray platform (BioProject PRJNA135933) during four seasons in four different areas of the Venice Lagoon. Finally, pharmaceuticals also constitute a new emerging class of environmental contaminants continuously released in aquatic environments. Among them, the effect of exposure to ibuprofen has been already tested on *R*. *philippinarum* using microarrays, revealing an alteration of several molecular pathways, including arachidonic acid metabolism, apoptosis, peroxisomal proliferator-activated receptors, and nuclear factor-kappa B, helping elucidate the putative mechanisms of action of ibuprofen in non-model species [6].

### 4.4. Proteomic Biomonitoring of Harmful Marine Compounds

Proteomic approaches are a valuable complement of transcriptomic studies in bivalves and can support the identification of new biomarkers of xenobiotic toxicity [4]. For instance, a consistent alteration of 13 proteins was found along a metal contamination gradient in *Crassostrea hongkongensis*, supporting their potential application as diagnostic tools for the assessment of metal pollution in environmental monitoring programs [109]. Moreover, putative toxicity biomarkers (including stress-related proteins and novel proteins families) for emerging pollutants, ionic Ag and Ag nanoparticles were also recently identified in *M*. *galloprovincialis* [110]. The proteomic approach was also validated in studies investigating the response to salinity stress, climate changes and accumulation of toxins of algal and cyanobacterial origin in different *Mytilus* species [111,112,113,114] as well as the response to heat stress in the salt marsh mussel *Geukensia demissa* [115]. Overall, the synergistic combination of different omic approaches has been decisive in elucidating the complex mechanisms underlying the adaptive response of marine bivalves to environmental changes. The results obtained so far often highlight interesting and surprising evidence that could not have been detected by common research approaches without time-consuming, complicated and expensive assays.

## 5. Conclusions

The great relevance of marine invertebrates makes the lack of bivalve model organisms puzzling. Still, the information reviewed throughout this work supports two bivalve molluscs, the Pacific oyster and the blue mussel, as upcoming model organisms. This notion is sustained by progressive omic characterizations of these organisms during the last decade, unleashing many potential applications most notably for pollution biomonitoring. Yet, the implications of bivalve omics for other research fields are still unexplored. Given the high bioaccumulation rates associated with the filtering capacity of bivalves and their relative tolerance to xenobiotics, it would not be surprising if new useful marine compounds, proteins or whole metabolic pathways could be discovered as a result of the omic analysis of these organisms. Although the possibilities promise to be endless, the development of further applications is still hampered by the early stage of development of omic technologies and associated computational methods of data analysis in bivalve molluscs, definitely far behind traditional model organisms. In such a scenario, and given the nature of the omic data, the advance in the integrative knowledge of bivalves will require coordination and transfer of knowledge across researchers sharing complementary goals. Indeed, the release of web accessible databases containing processed and reviewed results (not simply raw data) seems the best way to consolidate the omic characterization of bivalve molluscs. Altogether, great expectations are placed on future bivalve omics as it pertains to life sciences, environmental sciences and aquaculture livestock managing. Nonetheless, there is undoubtedly a long road ahead to obtain a truly holistic understanding of the basic features displayed by the different bivalve omes.
